# On a vectorcardiographic machine-learning approach to cardiology triage: target definition, calibration, and clinical utility

**DOI:** 10.1016/j.clinsp.2026.101070

**Published:** 2026-07-29

**Authors:** Juan Sebastián Therán León

**Affiliations:** Family Medicine Program, Universidad de Santander (UDES), Bucaramanga, Santander, Colombia

Dear Editor,

I read with interest the study by da Costa et al.,^1^ which examines whether Vectorcardiographic (VCG) markers of Global Electrical Heterogeneity (GEH), extracted from the standard 12-lead electrocardiogram and integrated into an explainable XGBoost model, can support triage in a referred cardiology population. The authors deserve credit for several methodological choices: the use of GEH derived from a routine examination, the dual gain- and SHAP-based explainability, the transparent best- and worst-case imputation analysis for incomplete follow-up, and a candid acknowledgement of the limited specificity. I offer the following observations in a constructive spirit, anchored in TRIPOD+AI and PROBAST.^2^^,^^3^

My principal concern is one of construct validity. The stated objective is to identify patients requiring tertiary care, an appropriateness-of-level construct, whereas the label actually modelled is the occurrence of death or a new nonfatal cardiovascular event (stroke, myocardial infarction, percutaneous coronary intervention, cardiac surgery) over 6–15 months. These are not the same target. The cohort had already been referred to, and was attending, a tertiary/quaternary ambulatory; predicting subsequent events within an already-referred population is a prognostic task, not an adjudication of who needed that referral. No reference standard for appropriate level of care was defined, and therefore the triage interpretation does not follow directly from the data. I would encourage the authors to reframe the work explicitly as outcome prediction, accompanied by a clear intended-use statement, as TRIPOD+AI recommends.^2^

A second concern is the absence of calibration. The report provides discrimination (AUC = 0.676) and threshold-based operating characteristics, but no calibration assessment ‒ neither a calibration plot nor calibration-in-the-large and calibration slope. For any model intended to inform referral, discrimination without calibration is insufficient: a poorly calibrated model can systematically over- or under-estimate risk and thereby misdirect scarce resources even when the rank-ordering of patients is preserved.^4^ Calibration is indispensable for the resource-allocation use the authors envisage.

Third, and most consequential for the clinical claim, no decision-curve analysis or net-benefit assessment is presented. The manuscript advances a cost-effectiveness rationale and explicitly weighs the harm of over-referral, yet utility is never quantified against the relevant default strategies (refer all, refer none, or a simple rule based on prior cardiac events). This omission is quantitatively material. At the reported operating point (sensitivity 94.1%, specificity 30.8%) and the cohort event prevalence (51/274; 18.6%), the rule classifies roughly 74% of an already-referred cohort as still requiring referral and screens out only about 26%, at a positive predictive value near 0.24 and at the cost of missing approximately 6% of the patients who experienced events. Whether this represents net benefit over referral guided by prior myocardial infarction or prior percutaneous coronary intervention alone ‒ both strongly associated with the outcome (p < 0.001) ‒ is precisely the question decision-curve analysis was designed to answer,^5^ and it remains untested.

Fourth, several features of the analysis raise the risk of optimistic bias, which PROBAST would flag in the analysis domain.^3^ First, selecting, from 50 trained instances, the single instance with the highest AUC as the representative model is a selection procedure that can inflate apparent performance unless the selection set is strictly independent of the held-out test set; the partition on which the maximum-AUC instance was chosen should be stated. Second, five-fold cross-validation performed without stratification with only 51 positive cases risks folds containing very few or no events, destabilising hyperparameter tuning. Third, the hybrid over- and under-sampling (ROSE) must be confined to the training partition within each resampling fold; if applied before partitioning, it introduces leakage. The sequence relative to the cross-validation split is not fully specified and warrants clarification.

Fifth, the development sample is modest for the feature space. Fifty-one events against a candidate set spanning standard ECG intervals, more than ten GEH parameters, and several clinical risk factors yields an events-per-candidate-predictor ratio well below contemporary recommendations,^6^ and the single-centre, four-month (May–August 2017) sampling frame further constrains generalizability. No external or temporal validation is provided; appropriate internal-external or external validation would substantially strengthen the inference.^7^ Relatedly, the combined model improved discrimination only marginally over the risk-factors-only model (AUC 0.676 vs. 0.625); whether this gain justifies the added complexity, and whether a parsimonious logistic rule would perform comparably, is worth examining, particularly given the limited sample.^8^

Finally, the deployment narrative deserves tempering. The automatable, scalable, low-cost screening framing ‒ and the explicit invocation of primary care, ambulances, and telemedicine ‒ sits uneasily with a pipeline in which a cardiology specialist manually marked the baseline and peaks across at least three cardiac cycles to compute GEH. As executed, the method is specialist-dependent; its portability to non-specialist settings is therefore an aspiration rather than a demonstrated property, and would require validation of an automated delineation step.

None of these points diminishes the prognostic promise of GEH, which is well grounded in prior cohort work. They are, however, addressable, and the contribution would be considerably strengthened by (i) Reframing the target as prognostic prediction with an explicit intended-use statement, (ii) Reporting calibration and decision-curve analysis, (iii) Prespecified, leakage-free resampling nested within cross-validation together with clarification of the instance-selection step, and (iv) External or temporal validation incorporating an automated GEH pipeline, all reported in line with TRIPOD+AI. I thank the authors for a stimulating contribution to a clinically important problem.

## Authorship

The author conceived, drafted, critically revised, and approved this correspondence and is accountable for all aspects of the work.

## Data availability statement

This comment did not generate new data; all data discussed are available in the cited article.

## Declaration of generative AI and AI-assisted technologies in the manuscript preparation process

During the preparation of this work the author used Claude (Anthropic) in order to assist with drafting and language editing of this correspondence. After using this tool, the author reviewed and edited the content as needed and takes full responsibility for the content of the publication.

## Funding

This research did not receive any specific grant from funding agencies in the public, commercial, or not-for-profit sectors.

## Declaration of competing interest

The authors declare no conflicts of interest.
